# Uncovering the genetic basis of crown rust resistance in a northern-by-southern oat biparental population

**DOI:** 10.1371/journal.pone.0351420

**Published:** 2026-06-24

**Authors:** Suraj Sapkota, Flavia Furlan, Stephen A. Harrison, Noah DeWitt, Md Ali Babar, Belayneh Admassu Yimer, Rawnaq N. Chowdhury, Kathy Esvelt Klos

**Affiliations:** 1 Oak Ridge Institute for Science and Education (ORISE) Research Participant, Small Grains and Potato Germplasm Research Unit, Aberdeen, Idaho, United States of America; 2 LSU AgCenter – School of Plant, Environmental and Soil Sciences, Baton Rouge, Louisiana, United States of America; 3 Department of Agronomy, University of Florida, Gainesville, Florida, United States of America; 4 Small Grains and Potato Germplasm Research Unit, USDA-ARS, Aberdeen, Idaho, United States of America; Brigham Young University, UNITED STATES OF AMERICA

## Abstract

Crown rust, caused by the fungal pathogen *Puccinia coronata* f. sp. *avenae* (*Pca*), is a destructive foliar disease of oat. Use of host resistance is the preferred method of disease management. However, frequent emergence of new *Pca* races has hindered crown rust management in oat. Identification and deployment of non-race-specific genetic resistance will aid oat breeding efforts to develop germplasm with durable crown rust resistance. To map and characterize crown rust resistance, a recombinant inbred line (RIL) population was developed from a cross between LA07065_SBSBSB_32-2 and CDC Dancer and evaluated for crown rust reaction in four field environments for two years. The RIL population was genotyped using the oat 6K Infinium iSelect single nucleotide polymorphism (SNP) array and a total of 956 polymorphic SNP markers were used to construct the linkage map. Quantitative trait loci (QTL) mapping detected a total of six QTL on chromosomes 2D, 4A, 7A, 7C, and 7D influencing crown rust resistance. Two of these QTL were validated using genotype-phenotype association analysis in an independent set of oat lines. The identified QTL demonstrated additive effects on crown rust resistance within the RIL population. A major QTL on chromosome 7C, *QPca-ars-7C*, was detected consistently across environments and explained up to 16.54% of the phenotypic variation. Markers linked to *QPca-ars-7C,* and other QTL detected in this study have the potential to be used in marker-assisted selection for crown rust resistance.

## Introduction

Oat (*Avena sativa* L.), a staple crop, holds an important position in global cereal production, ranking seventh among cereals in terms of production [1]. Its versatile applications as forage, in the cosmetic industry, and as a valuable component of a healthy diet have contributed to its popularity. Increased awareness among consumers about its nutritional value, and cardiovascular health benefits have elevated the importance of oat production worldwide [2]. However, oat production has been impacted by many biotic and abiotic stresses. Crown rust, caused by the fungal pathogen *Puccinia coronata* f. sp. *avenae* (*Pca*), is a destructive foliar disease of oat. Crown rust infection negatively affects oat yield, groat percentage, and straw strength [3,4].

Several management strategies that include, but are not limited to, genetic resistance, chemical (fungicide) control, eradication of the alternate host, and cultural practices that reduce the primary inoculum and rate of infection can be utilized to control crown rust [4,5]. Use of fungicides is not highly recommended because of negative environmental impacts, high cost of application, and likely development of fungicide resistance in rusts pathogen populations [1,4]. Genetic resistance is the most effective and preferred method to control crown rust.

The genetics of crown rust resistance in oat is broadly classified into seedling and adult plant resistance (APR). Seedling resistance, also known as all stage resistance, is race-specific and provides a high level of resistance. However, a major risk of using single-gene race-specific resistance in cultivar development is that pathogen evolution can overcome the resistance before the breeder can identify and deploy a replacement. The effectiveness of race-specific resistance typically lasts from 3 to 7 years [6]. More than 100 crown rust resistance genes (*Pc* genes) have been discovered [4,7] and most of them confer race-specific resistance. Only a few have been genetically mapped including *Pc38* and *Pc39* [8], *PcKm/Pc45* [9,10], *Pc48* [8], *Pc50-5* [11], *Pc53* [12], *Pc54* [13], *Pc58* [5,14], *Pc68* [15], *Pc71* [16], *Pc91* [17], *Pc94* [18], *Pc96* [19] and *Pc98* [20]. The complexity of the oat genome, with highly repetitive sequences, makes it challenging to genetically map and characterize *Pc* genes [21].

APR is conferred by multiple genes with minor effects that slow pathogen colonization and sporulation at the adult plant stage, thus reducing epidemic severity and limiting impact on crop yield and quality. Under field evaluations at the adult plant stage, researchers typically record rust severity data to study the genetics of none-race specific resistance; however, since plants are exposed to a diverse mixture of *Pca* races, it is also important to record the possible presence of a hypersensitive reaction, which indicates the presence of race-specific resistance in addition to none-race specific resistance [4,22]. Selection pressure on *Pca* populations from APR is lower compared to race-specific resistance making it more durable than single gene resistance [1]. Out of the catalogued *Pc* genes, only some have been definitively confirmed to confer APR [12,23–25]. Among these, the precise genomic position of only a few APR-associated QTL have been mapped on the oat reference genome [26–29].

The objective of most oat breeding programs worldwide is the release of cultivars with high yield/quality potential and disease resistance, especially to crown rust disease. *P. coronata* f. sp. *avenae* poses a persistent challenge in oat breeding in North America due to its potential for rapid mutation generating new races that overcome existing resistance mechanisms [4,30]. Thus, the pursuit of oat cultivars resistant to crown rust remains pivotal in oat breeding. Most southern U.S. oat germplasm in advanced trials has some level of crown rust resistance, but the type of resistance, and specific genes involved are unknown. The objectives of this study were to dissect the genetics of crown rust resistance in a southern oat breeding line and to identify QTL influencing crown rust resistance. This knowledge enables breeders to effectively implement resistance sources in the breeding programs, leading to the development of superior oat cultivars with enhanced crown rust resistance.

## Materials and methods

### Plant Materials

A recombinant inbred line (RIL) population, consisting of 124 lines, was generated through a cross between the spring breeding line LA07065_SBSBSB_32-2 and a spring oat cultivar CDC Dancer. This population, referred to as AIA1405 hereafter, was developed at the Small Grains and Potato Germplasm Research Facility of the USDA-ARS in Aberdeen, Idaho. The CDC Dancer cultivar originated from the University of Saskatchewan in Canada, with a pedigree of OT344 x OT269. It showed intermediate reaction to crown rust. The LA07065_SBSBSB_32-2 was developed by Louisiana State University (LSU) with the pedigree of TAM O-405 x UFRGS 028152-1 and exhibits crown rust resistance at the adult plant stage.

### Field tests for crown rust assessment

The AIA1405 population and parental lines were evaluated for adult pant crown rust reaction at the Louisiana State University experimental station at Baton Rouge, LA in 2020–2021 (BR21) and 2021–2022 (BR22), at Winnsboro, LA in 2021–2022 (WIN22), and at the University of Florida – Citra station in 2021–2022 (CFL22) growing seasons. The plant materials were planted in 11/20/2020, 11/23/2021, 11/03/2021, and 12/08/2021 at BR21, BR22, WIN22, and CFL22, environments, respectively. A randomized complete block design was used with two replications in the 2020–2021 season, and with three replications in the 2021–2022 growing season at all locations. To facilitate natural infection, a susceptible cultivar named ‘Brooks’ was used as a disease spreader and planted after every 10 rows. Disease severity was assessed at the milk to dough grain filling stage on a whole plot basis using the modified Cobb Scale, which ranges from 0 to 100% [31]. The infection response (IR) was categorized based on plant responses, as follows: resistant (necrotic flecks – R), moderately resistant (small uredinia surrounded by necrosis or chlorosis – MR), moderately susceptible (medium to large uredinia surrounded by chlorosis – MS), and susceptible (large uredinia with no chlorosis or necrosis – S) [17]. The categorical score of IR was later converted into numerical value (R = 0.2, MR = 0.4, MS = 0.8, and S = 1.0) and used in the subsequent genetic analysis [32]. Combinations of IRs were also possible when the symptoms observed were intermediate between IR categories.

### Statistical analysis of phenotypic data

Summary statistics of phenotypic data from all location-years were visualized using JMP Pro 17 software (SAS Institute, Cary, NC). Analysis of variance (ANOVA) was performed using the proc GLM procedure in SAS software version 9.4 (SAS Institute, Cary, NC). Least-square means (LS mean) were calculated separately for each environment and across all environments and used in downstream analysis. Distribution of the phenotypic data was visualized using the ‘ggplot’ in R [33]. Pearson correlation coefficients (*r*) for crown rust severity and IR within and among environments were calculated using the pairs.panel function in R package “psych” [34] to determine the consistency of crown rust response across environments. Broad sense heritability (*H*^2^) was calculated separately for individual environments and across all environments using Equations 1 and 2, respectively.


$$\begin{array}{c} H^{\text{2}}=\;\frac{\overset{\text{2}}{\underset{G}{\sigma }}}{\overset{\text{2}}{\underset{G\;}{\sigma }}+\;\overset{\text{2}}{\underset{\frac{e}{r}}{\sigma }}\;} \end{array}$$ (1)

$$\begin{array}{c} H^{\text{2}}=\;\frac{\overset{\text{2}}{\underset{G}{\sigma }}}{\overset{\text{2}}{\underset{G\;}{\sigma }}+\;\overset{\text{2}}{\underset{\frac{GE\;}{e}}{\sigma }}+\;\overset{\text{2}}{\underset{\frac{e}{er}}{\sigma }}\;} \end{array}$$ (2)

where $\overset{\text{2}}{\underset{G}{\sigma }}$ is the genotypic variance, $\overset{\text{2}}{\underset{GE}{\sigma }}$ is the genotype by environment interaction variance, $\overset{\text{2}}{\underset{e}{\sigma }}$ is the error variance, and *e* and *r* denote the number environments and replications, respectively. PROC VARCOMP in SAS 9.4 software was used to estimate variance components.

### Genotyping, linkage map construction, and QTL analysis

DNA was extracted using the cetyltrimethylammonium bromide protocol of [35] with modification including use of a FastPrep homogenizer (MP Biomedical) for 5 minutes at 25 strokes/second to homogenize tissue. The high-quality DNA from parental lines and RILs was genotyped using the oat 6K Infinium iSelect SNP array that is manufactured by Illumina (San Diego, CA) at the Cereal Crops Research Unit of ARS-USDA in Fargo, ND. SNP markers were called automatically using the Genome Studio 2.0 software (Illumina, San Diego, CA, 2016) and were manually assessed for call accuracy. Polymorphic markers that had less than 20% missing data and >0.05 minor allele frequency (MAF) were selected and used for linkage map construction. Of 4,852 SNP markers, 956 markers passed the quality control parameters. Information about the chromosomal location and physical position of SNP markers were obtained from *A. sativa* OT3098 v2 (PepsiCo, https://wheat.pw.usda.gov/jb?data=/ggds/oat-ot3098v2-pepsico).

Linkage map construction and QTL analysis were performed using QTL IciMapping v4.2 software [36]. For linkage map construction, the MAP function was used with the default parameters including 3.0 LOD threshold, k-optimality algorithm, and 5-marker window size. A genetic distance of 50 cM between the adjacent markers was used as a threshold to determine the linkage groups. The graphical representation of the genetic map was generated using MapChart [37]. QTL analysis was performed using the BIP (QTL mapping in bi-parental populations) function of the inclusive composite interval mapping of additive (ICIM-ADD) QTL method. After performing a 1000-permutation test, a logarithm of odds (LOD) threshold of 3.0 was set to declare significant QTL. LOD score of 3, which corresponds to a chance of 1 to 1000 that observed linkage is false position, is a commonly used statistical cutoff to declare significant QTL in linkage mapping [38]. The QTL IciMapping software by default provided the proportion of the phenotypic variance explained by each QTL and the magnitude of their additive effects. The additive effect of QTL was determined by calculating the mean severity and IR of RILs with resistance and susceptible alleles and comparing them using the Tukey’s HSD test (p < 0.05).

### Genotype-phenotype association analysis

To further investigate the presence and effect of the QTL detected in this study, two sets of oat consisting of 119 and 400 lines were selected from the Collaborative Oat Research Enterprise (CORE) panel [39]. The119 oat lines (southern panel) were from the southern US oat subpopulation and included lines developed by the LSU breeding program. The 400 oat lines (northern panel) were from the northern US oat subpopulation developed by various breeding programs for spring planted oat. The CORE panel was previously genotyped using genotyping-by-sequencing (GBS) [40]. SNPs with MAF of ≥ 0.05 and missing data of < 0.2 were retained and used for the genotype-phenotype association analysis. For these analyses, markers were also removed if there were fewer than 10 rare allele homozygotes within subpopulation. After quality filtering, a total of 437 and 413 SNP markers were retained for the analysis in the southern and northern panels, respectively. Crown rust field observations from Baton Rouge, LA and Castroville, TX were used for the southern panel whereas the observations from Fargo, ND, St. Paul, MN, Winnipeg, MB, and Ottawa, ON were used for the northern panel [7].

The 95% confidence interval around each QTL detected in the AIA1405 RIL population was established as the 1-LOD region from the peak position as described by Klos et al. [41]. The corresponding regions on the oat consensus map were determined based on the position of shared markers within the QTL regions [40]. All SNPs located within the QTL regions on the consensus map were identified, but only those present in the genotypic data of the southern and northern panels were retained for analysis. Based on the population structure analysis of Klos et al. [39], association between marker genotype and phenotype was conducted using a mixed linear model (MLM) in TASSEL v5.2.8 [42]. The Bonferroni correction method was chosen to reduce the false positive rate as a result of multiple testing and *p* ≤ 0.000114 and *p* ≤ 0.000121 at *α* = 0.05 was taken as a threshold to declare significant genotype-phenotype association in the southern and northern panels, respectively.

## Results

### Phenotypic analysis of RIL population

**Fig 1:**
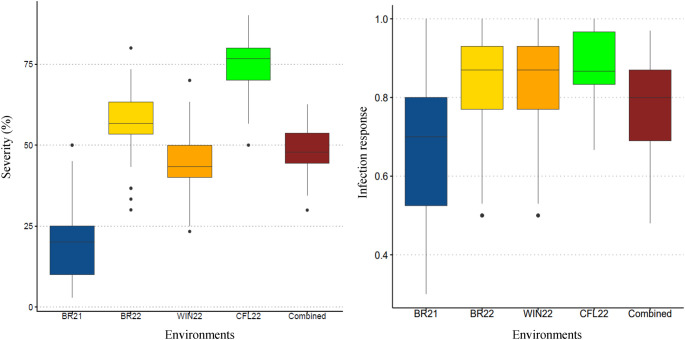
Boxplot showing the distribution of crown rust severity and infection response (IR) data in the AIA1405 RIL population.BR21, Baton Rouge 2021; BR22, Baton Rouge 2022; WIN22, Winnsboro 2022; CFL22, Citra Floria 2022; Combined, data across all location years was combined.

In the BR21 environment (location and year), the disease pressure was relatively low, and both parents showed resistant reactions with disease severity less than 10%. The disease pressure in BR21 was low with most lines showing LS means of severity <30%; however, all locations in the following year experienced a substantial increase in disease pressure (Fig 1).

LA07065_SBSBSB_32-2 displayed moderately resistant (MR) to moderately susceptible (MS) crown rust reactions with disease severity ranging from 30-50%. CDC Dancer showed MS to susceptible (S) reactions with severity ranging from 50-60%. The range of LS means of crown rust severity in BR22, WIN22, and CFL22 was 30–80%, 23.33-70%, and 50–90%, respectively, in the RIL population. The distribution of LS means of crown rust severity and IR in each single environment and across all environments is presented in Fig 1. RILs differed significantly (*p* < 0.05) for their reaction to crown rust in all environments (location-years) except IR at CFL22 (S1 Table). IR phenotypes from CFL22 were not used in QTL mapping. When the phenotypic data across all environments was combined, ANOVA detected significant (*p* < 0.0001) variation among RILs, environments, and line × environment interactions (S1 Table). In all environments, transgressive segregation in the AIA1405 population for reaction to crown rust was observed, indicating that both parents contributed to the resistance responses.

### Correlation and heritability analysis

**Fig 2:**
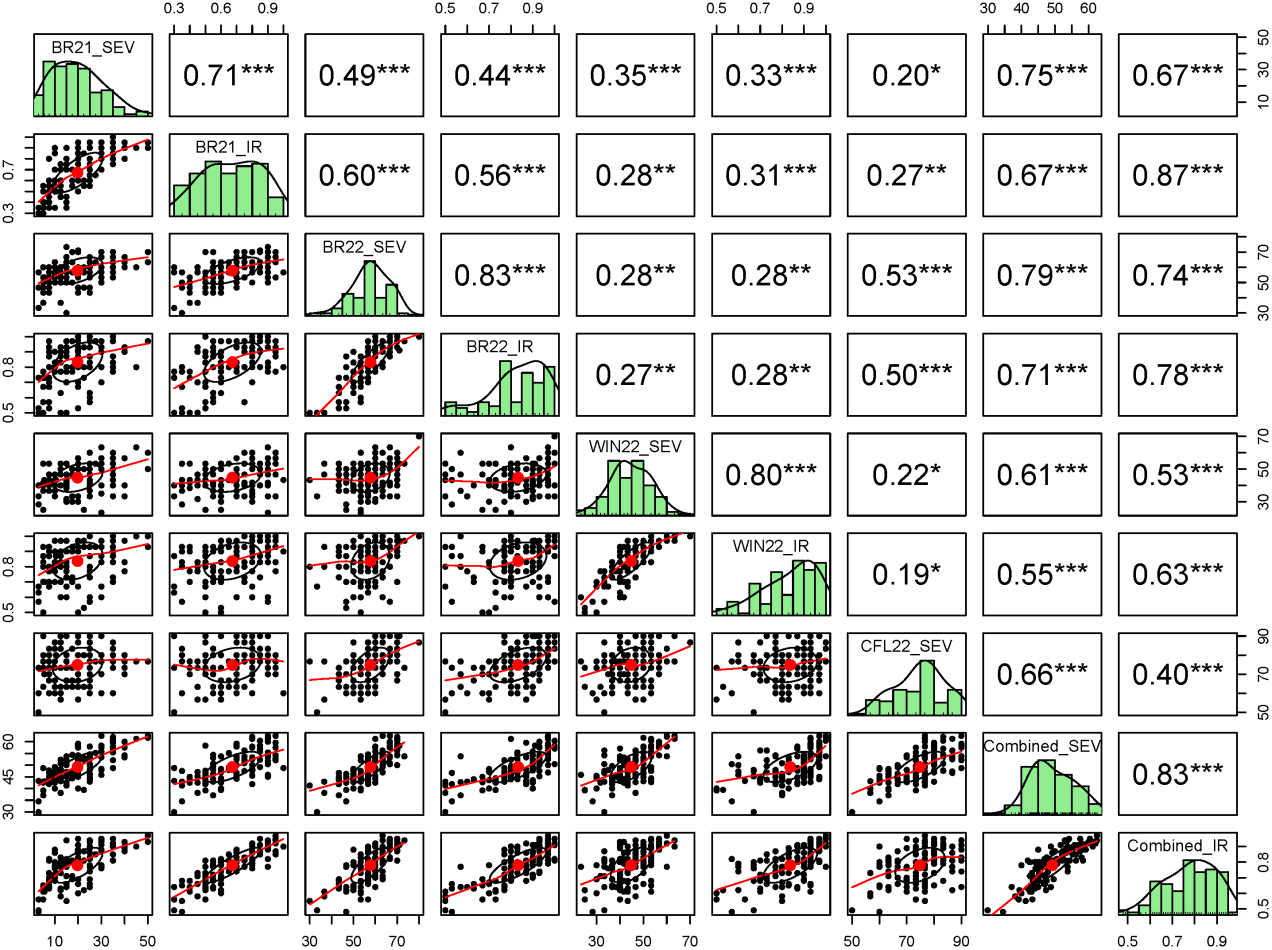
Correlation analysis of crown rust severity (SEV) and infection response (IR) data of AIA1405 recombinant inbred line (RIL) population collected from four field environments (BR21, Baton Rouge 2020-2021; BR22, Baton Rouge 2021-2022; WIN22, Winnsboro 2021-2022; CFL22, Citra Florida 2021-2022; and Combined, data across all location years was combined). The diagonal plots show the frequency distribution of phenotypic data. The panel above and below the diagonal represents Pearson’s correlation coefficient and scatter plots, respectively. *, ** and *** depict significant at 0.05, 0.01 and 0.001 probabilities levels, respectively.

As expected, there was a significant positive correlation between crown rust severity and IR data within environments and when the data across all environments was combined (Fig 2). The crown rust phenotypic data were significantly correlated across environments with values ranging from r = 0.19 (*p* < 0.05) to 0.83 (*p* < 0.001) indicating a stable crown rust response across environments (Fig 2). Similarly, when the data across environments was combined, the severity and IR data were highly correlated with r = 0.83 (*p* < 0.001). *H*^2^ estimates for both crown rust severity and IR were 0.69 when the data across environments was combined, indicting that crown rust resistance in this population was highly heritable (S2 Table).*H*^2^ estimates in individual environments ranged from 0.29 to 0.83 (S2 Table). *H*^2^ estimates for crown rust severity and IR data were higher in BR22 environment followed by WIN22, BR21, and CFL22 indicating that the environmental effect on crown rust reaction was larger in CFL22 (S2 Table).

### Genotyping data and linkage maps

After quality control, 956 SNP markers were used for linkage map construction and QTL analysis. The distribution of SNP markers among the 21 oat chromosomes ranged from 8 markers on chromosome 3A to 83 markers on chromosomes 1D and 4C (S3 Table). The total length of the linkage map was 3,836.76 cM with an average distance of 4.01 cM between adjacent markers (S3 Table). Since a distance between adjacent markers >50 cM was observed on chromosomes 3A, 3D, 4D, 5D, 6A, and 7A, these chromosomes were split into two linkage groups (LGs) making a total of 27 LGs. Chromosome 4D (LG15), which harbored only 4 markers with a genetic distance of 8.69 cM, was the shortest linkage map whereas chromosome 1D (LG3), which harbored 83 markers with a genetic distance of 376.70, was the longest linkage map. The A-genome contained the greatest number of markers followed by the C-and D-genomes (S3 Table).

### Detection of crown rust resistance QTL

**Fig 3:**
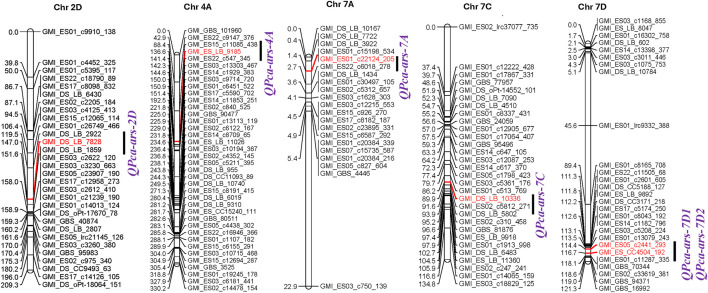
Linkage maps showing crown rust resistance QTL detected on chromosomes 2D, 4A, 7A, 7C, and 7D in the AIA1405 recombinant inbred line (RIL) population. The marker in the QTL peak position is highlighted in red font.

QTL mapping identified QTL on chromosomes 2D, 4A, 7A, 7C, and 7D influencing crown rust resistance in the AIA1405 RIL population (Table 1, Fig 3). Two QTL, *QPca-ars-2D* (resistance contributed by CDC Dancer) and *QPca-ars-4A* (resistance contributed by LA07065_SBSBSB_32–2), were detected when IR and severity data across environments was combined. The phenotypic variation they explained was 14.09% and 14.31% explained by *QPca-ars-2D* and *QPca-ars-4A*, respectively (Table 1). A QTL on chromosome 7A, *QPca-ars-7A*, was detected using IR data from BR22 and also when the severity data across all environments was combined. The allele that conferred resistance in *QPca-ars-7A was* derived from LA07065_SBSBSB_32–2. *QPca-ars-7A* had LOD score of 3.98 and 5.13 when BR22-IR and the combined-SEV dataset was used, respectively, and explained 6.79% of the phenotypic variation when combined severity data was used (Table 1). A QTL on chromosome 7C, *QPca-ars-7C*, was detected using severity and IR data from BR22 and the combined data from all environments. *QPca-ars-7C* was derived from LA07065_SBSBSB_32–2 and explained up to 16.54% of the phenotypic variation (Table 1). One or two QTL on chromosome 7D, *QPca-ars-7D1* and *QPca-ars-7D2*, were detected using IR and severity data, respectively. *QPca-ars-7D1* was derived from CDC Dancer with LOD value of 3.05 and explained 10.20% of the crown rust severity variation in BR22. The other QTL on chromosome 7D, *QPca-ars-7D2,* was derived from LA07065_SBSBSB_32–2 with a LOD value of 9.45 and explained 21.96% of the IR variation in WIN22 (Table 1). These overlapping LOD peaks may represent two tightly linked QTL or a single QLT with environmentally-dependent resistance alleles derived from both parents.

**Table 1:** Quantitative trait loci (QTL) detected for crown rust resistance in the AIA1405 recombinant inbred lines (RIL) population.

QTL	Env-trait^a^	Chr	Peak Marker	Peak Position (cM)^b^	Physical position (Mb)^c^	LOD^d^	PVE(%)^e^	Add^f^
*QPca-ars-2D*	Combined-IR	2D	GMI_DS_LB_7828	148.00	153.69	3.82	14.09	0.06
*QPca-ars-4A*	Combined-SEV	4A	GMI_ES_LB_9185	138.00	294.27	3.12	14.31	−3.30
*QPca-ars-7A*	BR22-IR	7A	GMI_ES01_c22124_205	2.00	68.62	5.13	8.55	−0.05
	Combined-SEV	7A	GMI_ES01_c22124_205	2.00	68.62	3.98	6.79	−2.35
*QPca-ars-7C*	Combined-SEV	7C	GMI_DS_LB_10336	90.00	67.44	5.33	10.45	−2.82
	Combined-IR	7C	GMI_DS_LB_10336	90.00	67.44	5.52	8.40	−0.04
	BR22-SEV	7C	GMI_DS_LB_10336	90.00	67.44	6.64	16.54	−3.78
	BR22-IR	7C	GMI_DS_LB_10336	90.00	67.44	6.31	12.08	−0.05
*QPca-ars-7D1*	WIN22-IR	7D	GMI_ES05_c2441_293	114.00	462.81	3.05	10.20	0.04
*QPca-ars-7D2*	BR22-SEV	7D	GMI_ES_CC4504_192	116.00	506.91	9.45	21.96	−4.46

^a^Env-trait, environment and trait combinations; BR22, Baton Rouge 2022; WIN22, Winnsboro 2022; Combined, data across all environments combined; SEV, severity; IR, infection response

^b^Genetic position of the QTL peak in centimorgan (cM)

^c^Physical position of the QTL peak marker in megabase pairs (Mb)

^d^LOD, Logarithm of odd value

^e^PVE, percentage of phenotypic variation explained by the QTL

^f^Add, additive effect. Negative and positive value indicates that the QTL was contributed by LA07065_SBSBSB_32–2 and CDC Dancer, respectively.

### Validation of QTL using genotype-phenotype association

Markers closest to LOD peaks in the AIA1405 RIL population (S4 Table) were not genotyped in the CORE association mapping panel, except GMI_ES_LB_9185 (*QPca-ars-4A*), GMI_ES02_c5812_271 (*QPca-ars-7C*), and GMI_ES_CC4504_192 (*QPca-ars-7D2*). The 95% confidence interval region of QTL detected in the AIA1405 RIL population and the corresponding region in the consensus map is presented in S4 Table. Out of all markers with genotypes available for the CORE panel, 836 markers were within the 95% confidence intervals of the QTL mapped in the AIA1405 RIL population. None of the markers available for the southern oat lines in the CORE were significantly associated with crown rust resistance at the Bonferroni correction threshold. Two markers within the 1-LOD confidence region of *QPca-ars-4A* were deemed suggestive of association with disease severity in the southern oat lines of the CORE at a less stringent threshold of p < 0.001 (S5 Table). In the larger panel of northern oat CORE lines, markers in the 1-LOD confidence regions of *QPca-ars-4A* and *QPca-ars-7C* were significantly associated with crown rust severity, while markers near *QPca-ars-7D1/ QPca-ars-7D2* were suggestive of association.

### QTL pyramiding effect

Three QTL, *QPca-ars-4A*, *QPca-ars-7A*, and *QPca-ars-7C*, influencing crown rust severity combined data and two QTL, *QPca-ars-2D* and *QPca-ars-7C*, influencing infection response combined data were further investigated to determine the effect of pyramiding resistance alleles. RILs were grouped based on the number of resistance alleles present, including a null group. Significant difference (*p* < 0.05) between the group of RILs with highest number of resistance alleles and the nulls was observed for both traits (S6 Table). As expected, pyramiding resistance alleles reduced crown rust severity and infection response (S2 Fig). The presence of one, two, and three resistance alleles reduced mean crown rust severity by 7.03, 14.40, and 26.86%, respectively, compared to the null group. Similarly, one and two resistance alleles reduced mean infection response by 5.95 and 15.48%, respectively, compared to the null group, suggesting an additive effect of these QTL (S6 Table).

## Discussion

The oat breeding line LA07065_SBSBSB_32–2 has consistently demonstrated a high level of crown rust resistance in germplasm evaluation nurseries. Therefore, to characterize the genetic architecture of crown rust resistance in the LA07065_SBSBSB_32–2, it was crossed with a susceptible oat cultivar CDC Dancer, and the resulting RIL population was evaluated under multiple field environments for crown rust reaction. As expected, given the large influence of environmental variation on crown rust disease, differing levels of infection were observed in the AIA1405 population across locations and over years. During the first year, crown rust infection at the Baton Rouge experiment station was low. However, in the second year of trial, a high disease pressure was observed at all locations (Fig 1). Although the diversity of *Pca* races in experimental sites has not been characterized in this study, the crown rust pathogen population in North America is extremely diverse due to its sexual reproduction on the alternate host *Rhamnus spp*. [1,6]. We believe that the diversity in virulence of prevalent *Pca* races across locations and over time also contributed to the differences in infection levels to some extent. Using inclusive composite interval mapping in the AIA1405 RIL population, six QTL on chromosomes 2D, 4A, 7A, 7C, and 7D influencing crown rust resistance were identified in this study (Table 1, Fig 3).

The efficiency of linkage-based QTL mapping greatly depends on the size and type of the mapping population, marker type, and the density of the molecular markers used to construct the linkage maps [43,44]. The linkage maps developed for six chromosomes in this study (3A, 3D, 4D, 5D, 6A and 7A) had poor marker coverage, which is likely because the genomes of the two parental lines are similar in those regions, and local SNPs on the Illumina 6K assay were not polymorphic. However, the linkage maps developed in this study are comparable, and in some cases, represented an improvement to previously reported linkage maps in oat. For example, Ociepa and Okon [45] reported linkage maps in oat that covered a total genetic distance of 24, 209.96 cM. Similarly, Ubert et al. [46] genotyped two oat RIL populations using the 6K Infinium iSelect SNP array, and constructed linkage maps consisting of 49 and 52 linkage groups, covering a total of 1450.5 and 1699.0 cM genetic distance, respectively. While the density of markers used to construct linkage maps in our study was sufficient for QTL analysis, it is unlikely to provide a high level of mapping resolution, and this should be considered in the interpretation of results and designing of future work.

Oat chromosome 2D, where *QPc-ars-2D* was detected in this study, harbors crown rust resistance genes including *Pc45/PcKM* and *Pc53* [1,10,12] and multiple QTL including *Qpc.CORE*.*08*.*1*, *Qpc.CORE*.*08*.*2*, *Qpc.CORE*.*08*.*3* and *QCr*.*cdl9-12D* [7,47]. Adamassu-Yimer et al. [12] reported that *Pc53* was mapped to within 1 cM location of *Pc45*/*PcKM* indicating that *Pc53* and *Pc45/PcKM* are likely allelic or linked. The markers closely linked to *QPc-ars-2D,* GMI_DS_LB_7828 is 18.3 cM away from GMI_ES02_c14533_567 (linked to *Pc53*), respectively, based on the oat consensus map of Chaffin et al. [48]. Klos et al. [7] mapped three QTL: *Qpc.CORE*.*08*.*1*, *Qpc.CORE*.*08*.*2*, and *Qpc.CORE*.*08*.*3* for crown rust resistance on chromosome 2D, and based on the oat consensus map, these three QTL are > 17 cM away from *QPc-ars-2D*. Babiker et al. [47] mapped *QCr.cdl9-12D* on chromosome 2D for crown rust resistance using a RIL population derived from a cross between Provena (susceptible parent) and CDC-Boyer (partially resistant parent). Based on the consensus map, markers GMI_ES15_c3200_563 (associated with *QCr.cdl9-12D*) and GMI_DS_LB_7828 (associated with *QPc-ars-2D*) are 10.9 cM apart. Given the overlapping evidence of genes/QTL on chromosome 2D, *QPc-ars-2D* may represent any one of the previously reported genes/QTL or a novel locus for crown rust resistance which warrants further investigation.

Two crown rust resistance genes, *Pc48* and *Pc98*, and multiple QTL including *QCr.cdl9-19A*, *QPc.CORE.20.1*, *QPc.CORE.20.2*, and *QPc.CORE.20.3*, were previously mapped on chromosome 4A [1,7,8,20,47] in the vicinity of *QPc-ars-4A*. Based on the consensus map of Chaffin et al. [48], SNP markers linked to *QPc-ars-4A* are more than 100 cM away from GMI_DS_LB_7494, a marker linked to *Pc98*, suggesting that *QPc-ars-4A* and *Pc98* are separate loci for crown rust resistance. *Pc48* was mapped on chromosome 4A using RFLP markers [1,8]. The markers linked to *Pc48* were located within 4–12 cM from *QPc-ars-4A* linked marker GMI_ES_LB_9185, based on the consensus map. Furthermore, the GrainGenes OT3098 v2 QTL/gene inventory data placed/mapped *Pc48* within the *QPc-ars-4A* region suggesting that they may represent the same locus for crown rust resistance. However, 65–100% of *Pca* isolates collected in the US and evaluated by the USDA-ARS Cereal Disease Lab between 2020 and 2023 were virulent on the *Pc48* differential line [49]. Although *Pc48* is defeated in many oat producing regions and fails to provide crown rust resistance [49], it is possible that *Pc48* was effective against some proportion of the naturally occurring crown rust in the environments used in this study, and contributed a small amount of resistance. Further research is needed to determine if *QPc-ars-4A* represents *Pc48* or a different locus for crown rust resistance.

Three closely linked crown rust resistance genes: *Pc46*, *Pc50*, and *Pc68* were reported on oat chromosome 7A [1,50,51]. However, Klos et al. [7] and Park et al. [1] have reported *Pc68* on chromosome 3D. Since the placement of these genes on the oat chromosome is not conclusive, comparison of the QTL detected on chromosome 7A in this study, *QPca-ars-7A,* with previously reported genes was difficult. A major QTL *QPca-ars-7C* influencing crown rust resistance was consistently detected on chromosome 7C in this study. Previous studies have reported crown rust resistance gene *Pc91* on chromosome 7C [1,52]. McCartney et al. [17] used DArT markers to map *Pc91*, and the alignment of the DArT markers associated with it indicated that *Pc91* was likely to be located either on chromosome 7C or 1A [1]. We believe that *QPca-ars-7C* represents a different locus than *Pc91*. Firstly, *Pc91* was derived from the hexaploid oat line ‘Amagalon (PI 497742)’, an interspecific cross between a tetraploid (*Avena magna*) and a diploid (*Avena longiglumis*) line [53]. LA07065_SBSBSB_32–2, donor of *QPca-ars-7C,* does not have ‘Amagalon’ in its pedigree indicating that *Pc91* and *QPca-ars-7C* likely represent separate loci for crown rust resistance. Secondly, haplotype analysis of LA07065_SBSBSB_32–2 and the *Pc91* differential line showed that the two did not share the same set of haplotypes indicating that *Pc91* gene is likely absent in LA07065_SBSBSB_32–2.

Both CDC Dancer and LA07065_SBSBSB_32–2 carry crown rust resistance QTL on an overlapping region of chromosome 7D, although that contributed by LA07065_SBSBSB_32–2 was more effective in these field trials. Although these QTL are placed to chromosome 7D based on the *A. sativa* OT3098 v2 sequence, the consensus map developed by Chaffin e al. [48,54] placed markers GMI_ES05_c2441_293 (*QPca-ars-7D1*) and GMI_ES_CC4504_192 (*QPca-ars-7D2)* to linkage groups Mrg33 (Chr2A) and Mrg02 (Chr7D), respectively. *QPca-ars-7D1* had an effect on crown rust IR suggesting race-specific resistance whereas *QPca-ars-7D2* had an effect on severity suggesting none race-specific resistance (Table 1). Crown rust resistance genes *Pc54*, *Pc58* complex (*Pc58a*, *Pc58b*, and *Pc58c*), *Pc38/Pc62/Pc63*, and multiple QTL including *QPc.Core.02*, *qPCRFd*, *Prq1a*, *Prq1b*, and *QCR.TxH*-*Mrg02* were previously mapped to chromosome 7D [5,7,8,13,50,55]. Of the three *Pc58* complex genes, *Pc58a* and *Pc58c* are located at 10.8 cM whereas *Pc58b* is located at 110.4 cM on the consensus map [5,13]. *QPca-ars-7D2,* located at 30.1 cM on consensus map, is unlikely to be part of the *Pc58* gene complex. Based on genetic analysis test, Harder et al. [55] reported that three crown rust resistance genes, *Pc38, Pc62,* and *Pc63,* were either linked or allelic. Admassu-Yimer et al. [13] mapped crown rust resistance gene *Pc54* by using two RIL populations on linkage group Mrg02 and reported that the location of *Pc54* gene overlapped with *Pc38/Pc62/Pc63* gene cluster*.* Based on the position of linked markers, *Pc54* spans 60–88 cM on the oat consensus map [13]. *QPca-ars-7D2,* located at 30.1 cM on the consensus map, is unlikely to be *Pc54* gene. The SNP marker associated with crown rust resistance QTL, *QPc.Core.02,* predictive of seedling resistance, is located at 28.1 cM on the consensus map [7]. Since *QPc.Core.02* and *QPca-ars-7D1* are located very close (2 cM apart) on chromosome 7D, they could represent the same locus. However, more genetic studies are required to dissect the actual relationships among them.

In addition to cross-referencing the QTL detected in the current study with those reported as detected in the CORE genome-wide association mapping panel, we performed a re-analysis of the CORE data using only those markers within the 1-LOD confidence interval of QTL mapped in this cross. This re-analysis differed from the original in two ways. First, because we detected overlapping or allelic QTL on Chromosome 7D derived from both the northern and southern parents of this cross, we chose to evaluate association within these groups of the CORE separately instead of using covariates to adjust for population stratification. Second, since we were evaluating only markers within the 1-LOD confidence regions of QTL mapped in the current study, we used two thresholds to determine statistical significance of association in the CORE. P-values <0.000121 and *p* ≤ 0.000114 (in the northern and southern oat subsets of the CORE, respectively) were considered statistically significant evidence of association based on the Bonferroni correction necessary to maintain an *α* = 0.05, while *p* ≤ 0.001 were considered suggestive of association in a region previously determined likely to influence crown rust reaction. CDC Dancer was included in the northern oat subset, while LA07065_SBSBSB_32–2 was not included in the CORE. Of the six QTLs identified in the AIA1405 RIL population, two QTLs, *QPca-ars-4A* and *QPca-ars-7C*, were detected in the northern panel of oat lines at the Bonferroni correction threshold (S5 Table). Although resistance alleles of both QTL were contributed by southern line, LA07065_SBSBSB_32–2 in the current cross (Table 1), they were detected in the northern oat Core subset using crown rust data from a single location year. The resistance alleles at these QTL may be present in oat germplasm developed by spring oat breeding programs in addition to fall-planted oat breeding programs. On average, northern oat CORE lines carrying the rare allele of both SNP markers, avgbs_cluster_13926.1.38 (*QPca-ars-4A*) and avgbs_10324.1.31 (*QPca-ars-7C*), exhibited lower crown rust severity compared to carriers of the common allele (S3 Fig). However, disease severity in that location year (Winnipeg, Canada 2011) was very light, suggesting caution when interpreting these results. When the less conservative *p*-value threshold of <0.001 was used to suggest statistical significance, three additional SNP markers were found to be associated with crown rust resistance in the CORE (S5 Table). SNP marker, avgbs_63110.1.59, located within the QTL region of *QPca-ars-7D1 and QPca-ars-7D2* was found to predict crown rust infection response data collected from St. Paul, MN (2011) and Fargo, ND (2010) for the spring planted CORE oat lines. Two additional SNP markers, avgbs_6K_80511.1.64 and avgbs_cluster_18331.1.38, in the *QPca-ars-4A* region were predictive of crown rust severity in Castroville, TX (2010) and Baton Rouge, LA (2011) of the southern oat lines at *p* < 0.001 (S5 Table).

Interestingly, the effect of variation in the *QPca-ars-4A* region appeared stronger in the spring planted oat lines of the CORE compared to effect within the AIA1405 population (Tables 1 and S5). This suggests potential benefits from greater use of this resistance in spring planted oat. Inability to validate all QTL identified in the AIA1405 RIL population using genotype-phenotype analysis in a subset of the CORE panel (southern and northern panels) indicates that either the linkage between markers and trait in the AIA1405 RIL population is false positive, or the effects of these QTL are too small to be detected in a genotype-phenotype association analysis. A small number of southern oat lines were used in the genotype-phenotype association analysis, which may lack a statistical power to detect all loci contributing to the crown rust resistance due to insufficient linkage disequilibrium between the markers and the QTL. Furthermore, since the AIA1405 RIL population was evaluated nine years after the southern and northern panels was evaluated, the race diversity of the crown rust pathogen may have changed, and different crown rust resistance genes/QTL may have been effective.

Molecular markers linked to QTLs could be of interest for plant breeders via marker-assisted selection (MAS) if they could differentiate parents with desirable and undesirable alleles with low false positive rate [51]. For MAS to be successful, QTL linked markers should be validated and effective in diverse genetic background. Of the markers linked to six QTL identified in this study, only three markers, GMI_ES_LB_9185 (*QPca-ars-4A*), GMI_ES02_c5812_271 (*QPca-ars-7C*), and GMI_ES_CC4504_192 (*QPca-ars-7D2*) segregated in Illumina 6K genotyping data of CORE lines. We observed lower frequency of resistance allele in the CORE panel for the GMI_ES_LB_9185 marker (16%) as compared to other two markers, GMI_ES02_c5812_271 (66%) and GMI_ES_CC4504_192 (66%), suggesting that GMI_ES_LB_9185 is a good candidate for MAS.

For non-race specific resistance, which is controlled by multiple QTL with minor effects, combining multiple QTL into a common genetic background could add up to an economically effective level of crown rust resistance. While the exact criteria to determine the economic level of crown rust resistance is not specified, previous studies have reported that yield loss in oat, due to each percent increase in crown rust severity is high. For example, Bissonnette et al. [56] reported that yield loss in oat cultivars Nobel and Ogle for each percent increase in crown rust severity was 56.7 and 46.0 kg/ha, respectively. Similarly, Bowen et al. [57] reported that depending upon cultivars and year, yield loss in oats could be 20–70 kg/ha for each unit increase in crown rust disease. Many studies further reported a significant negative correlation between crown rust infection and yield [58,59]. Overall, those studies have reported that the increase in crown rust disease, even in small percentage, could impact on oat yield and quality. In this study, when resistance allele from three QTL influencing crown rust severity was combined, the difference in crown rust severity between the groups of RILs containing three QTL and null was 14.70% (S6 Table). Out of 124 RILs evaluated for crown rust resistance in this study, six RILs possessed all three-resistance allele, and they have the potential to be used in breeding programs to develop durable crown rust resistance germplasm.

Breeding for crown rust resistance is a continuous effort since new *Pca* races are regularly emerging and can overcome effective genes. In this study, six QTL influencing crown rust resistance were identified including a major QTL, *QPca-ars-7C*, consistently detected across environments. Although comparison of QTL identified in this study with previously reported QTL/gene was done, further genetic tests are required to validate the novelty of identified QTL. However, regardless of whether the QTL detected in this study are novel or not, they represent a valuable source for crown rust resistance embedded in an elite oat cultivar and are ready for use in an oat breeding programs. SNP markers linked to the QTL have the potential to be converted into diagnostic markers that can be used in marker-assisted selection for crown rust resistance in oats.

## Supporting information

S1 Table Analysis of variance (ANOVA) of crown rust phenotypic data collected from four environments. Env/trait, environment and trait combination. BR21, Baton Rouge 2021; BR22, Baton Rouge 2022; WINN22, Winnsboro 2022, CFL22, Citra Floria 2022; combined, data across all environments was combined.
(PDF)

S2 Table Variance components and heritability calculation of the crown rust phenotypic data collected from AI1405 recombinant inbred line (RIL) population. (PDF)

S3 Table Distribution of molecular markers and marker density across linkage groups in AIA1405 recombinant inbred line (RIL) mapping population. (PDF)

S4 Table 95% confidence intervals on QTL identified in the AIA1405 RIL population and their corresponding region on the oat consensus map. (PDF)

S5 Table Marker-trait association results from analyses within fall-planted (primarily southern US) and spring-planted (northern) subsets of the CORE association mapping panel. (PDF)

S6 Table Effects of pyramiding selected resistance alleles on crown rust severity and infection response. (PDF)

S1 Fig Boxplots demonstrating the effect of QTLs on crown rust severity (left) and IR (right) in the AIA1405 RIL population using phenotypic data combined across environments. (PDF)

S2 Fig Distribution of crown rust severity data (Winnipeg 2011) of oat lines from the Collaborative Oat Research Enterprise (CORE) panel carrying AA/GG and CC/TT genotypes at avgbs_10324.1.31 (left) and avgbs_cluster_13926.1.38 (right) markers. (PDF)

S3 Fig Phenotypic differences between homozygous genotype classes associated with six QTL in the AIA1405 recombinant inbred line population. (PDF)

## References

[1] Park RF, Boshoff WHP, Cabral AL, Chong J, Martinelli JA, McMullen MS, et al. Breeding oat for resistance to the crown rust pathogen *Puccinia coronata* f. sp. avenae: achievements and prospects. Theor Appl Genet. 2022;135(11):3709–34. doi: 10.1007/s00122-022-04121-z 35665827 PMC9729147

[2] Gorash A, Armonienė R, Mitchell Fetch J, Liatukas Ž, Danytė V. Aspects in oat breeding: nutrition quality, nakedness and disease resistance, challenges and perspectives. Annals of Applied Biology. 2017;171(3):281–302. doi: 10.1111/aab.12375

[3] Doehlert DC, McMullen MS, Hammond JJ. Genotypic and environmental effects on grain yield and quality of oat grown in North Dakota. Crop Science. 2001;41(4):1066–72. doi: 10.2135/cropsci2001.4141066x

[4] Nazareno ES, Li F, Smith M, Park RF, Kianian SF, Figueroa M. P*uccinia coronata* f. sp. avenae: a threat to global oat production. Mol Plant Pathol. 2018;19(5):1047–60. doi: 10.1111/mpp.12608 28846186 PMC6638059

[5] Hoffman DL, Chong J, Jackson EW, Obert DE. Characterization and Mapping of a Crown Rust Resistance Gene Complex(Pc58) in TAM O‐301. Crop Sci. 2006;46(6):2630–5. doi: 10.2135/cropsci2006.01.0014

[6] Carson ML. Virulence frequencies in oat crown rust in the United States from 2001 through 2005. Plant Dis. 2008;92(3):379–84. doi: 10.1094/PDIS-92-3-0379 30769684

[7] Klos KE, Yimer BA, Babiker EM, Beattie AD, Bonman JM, Carson ML, et al. Genome-wide association mapping of crown rust resistance in oat elite germplasm. Plant Genome. 2017;10(2). doi: 10.3835/plantgenome2016.10.0107 28724060

[8] Wight CP, O’Donoughue LS, Chong J, Tinker NA, Molnar SJ. Discovery, localization, and sequence characterization of molecular markers for the crown rust resistance genes Pc38, Pc39, and Pc48 in cultivated oat (*Avena sativa* L.). Molecul Breed. 2004;14(4):349–61. doi: 10.1007/s11032-004-0148-z

[9] Gnanesh BN, McCartney CA, Eckstein PE, Mitchell Fetch JW, Menzies JG, Beattie AD. Genetic analysis and molecular mapping of a seedling crown rust resistance gene in oat. Theor Appl Genet. 2015;128(2):247–58. doi: 10.1007/s00122-014-2425-5 25433497

[10] Kebede AZ, Friesen-Enns J, Gnanesh BN, Menzies JG, Mitchell Fetch JW, Chong J, et al. Mapping oat crown rust resistance gene Pc45 confirms association with PcKM. G3 (Bethesda). 2019;9(2):505–11. doi: 10.1534/g3.118.200757 30554147 PMC6385968

[11] Toporowska J, Sowa S, Kilian A, Koroluk A, Paczos-Grzęda E. Discovery and chromosomal location a highly effective oat crown rust resistance gene Pc50-5. Int J Mol Sci. 2021;22(20):11183. doi: 10.3390/ijms222011183 34681841 PMC8540790

[12] Admassu-Yimer B, Bonman JM, Esvelt Klos K. Mapping of crown rust resistance gene Pc53 in oat (*Avena sativa*). PLoS One. 2018;13(12):e0209105. doi: 10.1371/journal.pone.0209105 30586454 PMC6306165

[13] Admassu-Yimer B, Klos KE, Griffiths I, Cowan A, Howarth C. Mapping of crown rust (*Puccinia coronata* f. sp. avenae) resistance gene Pc54 and a novel quantitative trait locus effective against powdery mildew (*Blumeria graminis* f. sp. avenae) in the oat (*Avena sativa*) Line Pc54. Phytopathology. 2022;112(6):1316–22. doi: 10.1094/PHYTO-10-21-0445-R 34982574

[14] Oliver RE, Tinker NA, Lazo GR, Chao S, Jellen EN, Carson ML, et al. SNP discovery and chromosome anchoring provide the first physically-anchored hexaploid oat map and reveal synteny with model species. PLoS One. 2013;8(3):e58068. doi: 10.1371/journal.pone.0058068 23533580 PMC3606164

[15] Chen G, Chong J, Gray M, Prashar S, Douglas Procunier J. Identification of single-nucleotide polymorphisms linked to resistance genePc68to crown rust in cultivated oat. Canadian Journal of Plant Pathology. 2006;28(2):214–22. doi: 10.1080/07060660609507289

[16] Bush AL, Wise RP. High-resolution mapping adjacent to the Pc71 crown-rust resistance locus in hexaploid oat. Molecular Breeding. 1998;4(1):13–21. doi: 10.1023/a:1009652222382

[17] McCartney CA, Stonehouse RG, Rossnagel BG, Eckstein PE, Scoles GJ, Zatorski T, et al. Mapping of the oat crown rust resistance gene Pc91. Theor Appl Genet. 2011;122(2):317–25. doi: 10.1007/s00122-010-1448-9 20862449

[18] Chong J, Reimer E, Somers D, Aung T, Penner GA. Development of sequence-characterized amplified region (SCAR) markers for resistance genePc94to crown rust in oat. Canadian Journal of Plant Pathology. 2004;26(1):89–96. doi: 10.1080/07060660409507118

[19] Abdullah S, Gordon T, Yimer BA, Paczos-Grzęda E, Harrison SA, Menzies JG, et al. Mapping and identification of molecular markers for the Pc96 gene conferring resistance to crown rust in oat. PLoS One. 2023;18(4):e0283769. 10.1371/journal.pone.0283769 PMC10079104 37023078

[20] Zhao J, Kebede AZ, Menzies JG, Paczos-Grzęda E, Chong J, Mitchell Fetch JW, et al. Chromosomal location of the crown rust resistance gene Pc98 in cultivated oat (*Avena sativa* L.). Theor Appl Genet. 2020;133(4):1109–22. doi: 10.1007/s00122-020-03535-x 31938813

[21] Yan H, Bekele WA, Wight CP, Peng Y, Langdon T, Latta RG, et al. High-density marker profiling confirms ancestral genomes of *Avena* species and identifies D-genome chromosomes of hexaploid oat. Theor Appl Genet. 2016;129(11):2133–49. doi: 10.1007/s00122-016-2762-7 27522358 PMC5069325

[22] Leonard KJ. Oat lines with effective adult plant resistance to crown rust. Plant Dis. 2002;86(6):593–8. doi: 10.1094/PDIS.2002.86.6.593 30823229

[23] Lin Y, Gnanesh BN, Chong J, Chen G, Beattie AD, Mitchell Fetch JW, et al. A major quantitative trait locus conferring adult plant partial resistance to crown rust in oat. BMC Plant Biol. 2014;14:250. doi: 10.1186/s12870-014-0250-2 25260759 PMC4181729

[24] Rines HW, Miller ME, Carson M, Chao S, Tiede T, Wiersma J, et al. Identification, introgression, and molecular marker genetic analysis and selection of a highly effective novel oat crown rust resistance from diploid oat, Avena strigosa. Theor Appl Genet. 2018;131(3):721–33. doi: 10.1007/s00122-017-3031-0 29222636

[25] Jackson E, Obert D, Menz M, Hu G, Bonman J. Qualitative and quantitative trait loci conditioning resistance to Puccinia coronata pathotypes NQMG and LGCG in the oat (*Avena sativ*a L.) cultivars Ogle and TAM O-301. Theoretical and Applied Genetics. 2008;116:517–27. 18193188 10.1007/s00122-007-0687-x

[26] Nazareno ES, Fiedler J, Miller ME, Figueroa M, Kianian SF. A reference-anchored oat linkage map reveals quantitative trait loci conferring adult plant resistance to crown rust (*Puccinia coronata* f. sp. avenae). Theor Appl Genet. 2022;135(10):3307–21. doi: 10.1007/s00122-022-04128-6 36029319

[27] Nguyen DT, Lewis D, Henningsen EC, Su Z, Mago R, Sperschneider J, et al. QTL mapping of oat crown rust resistance in Australian fields and identification of a seedling resistance locus in oat line GS7. Theor Appl Genet. 2026;139(1):41. doi: 10.1007/s00122-025-05145-x 41555018 PMC12815998

[28] Yimer BA, Gordon T, Bonman JM, Esvelt Klos K. Development and validation of a quantitative PCR assay method of assessing relative resistance of oat (*Avena sativa*) to crown rust (*Puccinia coronata* f. sp. avenae). Plant Pathology. 2019;68:669–77.

[29] Nazareno ES, Fiedler JD, Ardayfio NK, Miller ME, Figueroa M, Kianian SF. Genetic analysis and physical mapping of oat adult plant resistance loci against *Puccinia coronata* f. sp. avenae. Phytopathology. 2023;113(7):1307–16. doi: 10.1094/PHYTO-10-22-0395-R 36721375

[30] Figueroa M, Dodds PN, Henningsen EC. Evolution of virulence in rust fungi—multiple solutions to one problem. Current Opinion in Plant Biology. 2020;56:20–7. 32244171 10.1016/j.pbi.2020.02.007

[31] Peterson RF, Campbell AB, Hannah AE. A Diagrammatic scale for estimating rust intensity on leaves and stems of cereals. Can J Res. 1948;26c(5):496–500. doi: 10.1139/cjr48c-033

[32] Stubbs R, Prescott J, Saari E, Dubin H. Cereal Disease Methodology Manual. 1986.

[33] Team RDC. R: A Language and Environment for Statistical Computing. 2010.

[34] Revelle WR. Psych: Procedures for Personality and Psychological Research. 2017.

[35] Anderson JA, Ogihara Y, Sorrells ME, Tanksley SD. Development of a chromosomal arm map for wheat based on RFLP markers. Theor Appl Genet. 1992;83(8):1035–43. doi: 10.1007/BF00232969 24202932

[36] Meng L, Li H, Zhang L, Wang J. QTL IciMapping: Integrated software for genetic linkage map construction and quantitative trait locus mapping in biparental populations. The Crop Journal. 2015;3(3):269–83. doi: 10.1016/j.cj.2015.01.001

[37] Voorrips RE. MapChart: software for the graphical presentation of linkage maps and QTLs. J Hered. 2002;93(1):77–8. doi: 10.1093/jhered/93.1.77 12011185

[38] Lander ES, Botstein D. Mapping mendelian factors underlying quantitative traits using RFLP linkage maps. Genetics. 1989;121(1):185–99. doi: 10.1093/genetics/121.1.185 2563713 PMC1203601

[39] Esvelt Klos K, Huang Y-F, Bekele WA, Obert DE, Babiker E, Beattie AD, et al. Population Genomics Related to Adaptation in Elite Oat Germplasm. Plant Genome. 2016;9(2). doi: 10.3835/plantgenome2015.10.0103 27898836

[40] Bekele WA, Wight CP, Chao S, Howarth CJ, Tinker NA. Haplotype-based genotyping-by-sequencing in oat genome research. Plant Biotechnol J. 2018;16(8):1452–63. doi: 10.1111/pbi.12888 29345800 PMC6041447

[41] Esvelt Klos K, Gordon T, Bregitzer P, Hayes P, Chen XM, Del Blanco IA, et al. Barley Stripe rust resistance QTL: Development and validation of SNP markers for resistance to *Puccinia striiformis* f. sp. hordei. Phytopathology. 2016;106(11):1344–51. doi: 10.1094/PHYTO-09-15-0225-R 27213558

[42] Bradbury PJ, Zhang Z, Kroon DE, Casstevens TM, Ramdoss Y, Buckler ES. TASSEL: software for association mapping of complex traits in diverse samples. Bioinformatics. 2007;23(19):2633–5. doi: 10.1093/bioinformatics/btm308 17586829

[43] Chen Z, Wang B, Dong X, Liu H, Ren L, Chen J, et al. An ultra-high density bin-map for rapid QTL mapping for tassel and ear architecture in a large F₂ maize population. BMC Genomics. 2014;15(1):433. doi: 10.1186/1471-2164-15-433 24898122 PMC4059873

[44] Sapkota S, Mergoum M, Kumar A, Fiedler JD, Johnson J, Bland D, et al. A novel adult plant leaf rust resistance gene Lr2K38 mapped on wheat chromosome 1AL. Plant Genome. 2020;13(3):e20061. doi: 10.1002/tpg2.20061 33169935 PMC12807417

[45] Ociepa T, Okoń S. Chromosomal location of Pm12-A novel powdery mildew resistance gene from *Avena sterilis*. Genes (Basel). 2022;13(12):2409. doi: 10.3390/genes13122409 36553676 PMC9778159

[46] Ubert IP, Zimmer CM, Pellizzaro K, Federizzi LC, Nava IC. Genetics and molecular mapping of the naked grains in hexaploid oat. Euphytica. 2017;213(2). doi: 10.1007/s10681-017-1836-1

[47] Babiker EM, Gordon TC, Jackson EW, Chao S, Harrison SA, Carson ML, et al. Quantitative trait loci from two genotypes of oat (*Avena sativa*) conditioning resistance to *Puccinia coronata*. Phytopathology. 2015;105(2):239–45. doi: 10.1094/PHYTO-04-14-0114-R 25121640

[48] Chaffin AS, Huang Y-F, Smith S, Bekele WA, Babiker E, Gnanesh BN, et al. A consensus map in cultivated hexaploid oat reveals conserved grass synteny with substantial subgenome rearrangement. Plant Genome. 2016;9(2). doi: 10.3835/plantgenome2015.10.0102 27898818

[49] Moreau ELP, Riddle JM, Kianian SF. Virulence Dynamics of Puccinia coronata f. sp. avenae in 2023: Insights from the USDA’s Oat Crown Rust Survey. Plant Dis. 2025;109(9):1824–30. doi: 10.1094/PDIS-11-24-2408-SR 39970342

[50] Acevedo M, Jackson EW, Chong J, Rines HW, Harrison S, Bonman JM. Identification and validation of quantitative trait loci for partial resistance to crown rust in oat. Phytopathology. 2010;100(5):511–21. doi: 10.1094/PHYTO-100-5-0511 20373973

[51] Chowdhury RN, Nandety RS, Yimer BA, Fiedler J, Sapkota S, Klos KE. Development and accuracy assessment of molecular markers associated with crown rust resistance genes in oat. PLoS One. 2025;20(6):e0324826. doi: 10.1371/journal.pone.0324826 40549779 PMC12184994

[52] Gnanesh BN, Mitchell Fetch J, Menzies JG, Beattie AD, Eckstein PE, McCartney CA. Chromosome location and allele-specific PCR markers for marker-assisted selection of the oat crown rust resistance gene Pc91. Mol Breeding. 2013;32(3):679–86. doi: 10.1007/s11032-013-9900-6

[53] Rooney WL, Rines HW, Phillips RL. Identification of RFLP Markers Linked to Crown Rust Resistance Genes Pc 91 and Pc 92 in Oat. Crop Sci. 1994;34(4):940–4. doi: 10.2135/cropsci1994.0011183x003400040019x

[54] Jellen EN, Wight CP, Spannagl M, Blake VC, Chong J, Herrmann MH, et al. A uniform gene and chromosome nomenclature system for oat (*Avena* spp.). Crop Pasture Sci. 2024;75(1). doi: 10.1071/cp23247

[55] Harder DE, McKenzie RIH, Martens JW. Inheritance of crown rust resistance in three accessions of Avena sterilis. Can J Genet Cytol. 1980;22(1):27–33. doi: 10.1139/g80-005

[56] Bissonnette S, D’Arcy C, Pedersen W. Yield loss in two spring oat cultivars due to Puccinia coronata f. sp. avenae in the presence or absence of barley yellow dwarf virus. 1994.

[57] Bowen KL, Hagan AK, Pegues M, Jones J. Yield losses due to crown rust in winter oats in Alabama. Plant Health Progress. 2016;17(2):95–100. doi: 10.1094/php-rs-16-0016

[58] Holland JB, Munkvold GP. Genetic relationships of crown rust resistance, grain yield, test weight, and seed weight in oat. Crop Sci. 2001;41(4):1041–50. doi: 10.2135/cropsci2001.4141041x

[59] Singleton LL. Evaluation of oat crown rust disease parameters and yield in moderately resistant cultivars. Phytopathology. 1982;72(5):538. doi: 10.1094/phyto-72-538

