# Supplementary material for: Uncovering the genetic basis of crown rust resistance in a northern-by-southern oat biparental population
Source: PLoS One. 2026 Jun 24;21(6):e0351420. doi: 10.1371/journal.pone.0351420 (PMC13293447; doi:10.1371/journal.pone.0351420)
Supplement: S1 Table — Env/trait, environment and trait combination. BR21, Baton Rouge 2021; BR22, Baton Rouge 2022; WINN22, Winnsboro 2022, CFL22, Citra Floria 2022; combined, data across all environments was combined. (PDF) [file pone.0351420.s001.pdf]

Analysis of variance (ANOVA) of crown rust phenotypic data collected from four environments

**Env/traits[a]**	**Severity**						**Infection response (IR)**					
	Source	DF	Type III SS	Mean Square	F Value	Pr > F	Source	DF	Type III SS	Mean Square	F Value	Pr > F
**BR21**	Lines	124	28250	227.82258	1.56	0.0072	Lines	124	8.43664	0.06803742	1.96	0.0001
	Rep	1	1623.076	1623.076	11.08	0.0011	Rep	1	0.00576	0.00576	0.17	0.6841
	Source	DF	Type III SS	Mean Square	F Value	Pr > F	Source	DF	Type III SS	Mean Square	F Value	Pr > F
**BR22**	Lines	125	29050.13333	232.40107	6.02	<.0001	Lines	125	6.46050667	0.05168405	4	<.0001
	Rep	2	784.41905	392.20952	10.16	<.0	Rep	2	0.4494819	0.22474095	17.39	<.00
	Source	DF	Type III SS	Mean Square	F Value	Pr > F	Source	DF	Type III SS	Mean Square	F Value	Pr > F
**WINN22**	Line	125	28969.64286	231.75714	2.4	<.0001	Line	125	5.81322751	0.04650582	2.43	<.0001
	Rep	2	1795.63492	897.81746	9.3	0.0001	Rep	2	0.01529101	0.0076455	0.4	0.6707
	Source	DF	Type III SS	Mean Square	F Value	Pr > F	Source	DF	Type III SS	Mean Square	F Value	Pr > F
**CFL22**	Line	124	30489.06667	245.87957	1.41	0.0119	Line	124	4.27509333	0.03447656	1.13	0.2089
	Rep	2	2016.53333	1008.26667	5.78	0.0035	Rep	2	0.444	0.222	7.28	0.0008
	Source	DF	Type III SS	Mean Square	F Value	Pr > F	Source	DF	Type III SS	Mean Square	F Value	Pr > F
**Combined**	Line	125	51199.547	409.5964	4.58	<.0001	Line	125	12.7645188	0.10211615	5.03	<.0001
	Env	2	214999.7703	107499.8851	1203.05	<.0001	Env	2	4.89767324	2.44883662	120.52	<.0001
	Line*Env	249	36117.5018	145.0502	1.62	<.0001	Line*Env	249	8.61468077	0.03459711	1.7	<.0001

Env/trait, environment and trait combination. BR21, Baton Rouge 2021; BR22, Baton Rouge 2022; WINN22, Winnsboro 2022, CFL22, Citra Floria 2022; combined, data across all environments was combined

The number of lines used in ANOVA varied between environments but 124 lines with genotypic data were used for the genetic analysis.
