# Supplementary material for: Uncovering the genetic basis of crown rust resistance in a northern-by-southern oat biparental population
Source: PLoS One. 2026 Jun 24;21(6):e0351420. doi: 10.1371/journal.pone.0351420 (PMC13293447; doi:10.1371/journal.pone.0351420)
Supplement: S2 Table — (PDF) [file pone.0351420.s002.pdf]

Variance components and heritability calculation of the crown rust phenotpic data collected from AIA1405 recombinant inbred line (RIL) population

All environments combined						
Traits	Genotypic variance	Genotypie*Experiment variance	Error variance	Replication	Experiment	H^2
Severity	29.93201	13.30656	115.8283	3	4	0.698
Infection response (IR)	0.006377	0.0030705	0.02399	3	4	0.697

Single experiment					
Location year	Traits	Genotypic variance	Error variance	Replication	H^2
BR21	Severity	40.68781	146.44697	2	0.357188547
BR21	IR	0.01682	0.0344	2	0.49441505
BR22	Severity	64.88503	38.63485	3	0.834391273
BR22	IR	0.01294	0.01292	3	0.750289911
WIN22	Severity	45.06878	96.55079	3	0.583396679
WIN22	IR	0.0091616	0.01902	3	0.59100996
CFL22	Severity	23.82	174.39	3	0.29066504
