# Supplementary material for: Uncovering the genetic basis of crown rust resistance in a northern-by-southern oat biparental population
Source: PLoS One. 2026 Jun 24;21(6):e0351420. doi: 10.1371/journal.pone.0351420 (PMC13293447; doi:10.1371/journal.pone.0351420)
Supplement: S3 Table — (PDF) [file pone.0351420.s003.pdf]

Distribution of molecular markers and marker density across linkage groups in AIA1405 recombinant inbred line (RIL) mapping population

Linkage group (chr)	No. of Markers	Length (cM)	Marker density[a]
LG1 (1A)	50	366.75	7.34
LG2 (1C)	15	67.81	4.52
LG3 (1D)	83	376.70	4.54
LG4 (2A)	24	170.58	7.11
LG5 (2C)	34	84.44	2.48
LG6 (2D)	30	209.33	6.98
LG7 (3A)	4	78.17	19.54
LG8 (3A)	4	10.24	2.56
LG9 (3C)	20	109.98	5.50
LG10 (3D)	11	142.30	12.94
LG11 (3D)	6	80.77	13.46
LG12 (4A)	66	330.19	5.00
LG13 (4C)	83	113.14	1.36
LG14 (4D)	20	77.54	3.88
LG15 (4D)	4	8.69	2.17
LG16 (5A)	79	170.57	2.16
LG17 (5C)	46	368.04	8.00
LG18 (5D)	11	32.73	2.98
LG19 (5D)	25	110.72	4.43
LG20 (6A)	34	163.84	4.82
LG21 (6A)	9	42.42	4.71
LG22 (6C)	70	172.67	2.47
LG23 (6D)	31	124.59	4.02
LG24 (7A)	21	22.91	1.09
LG25 (7A)	60	145.48	2.42
LG26 (7C)	64	134.82	2.11
LG27 (7D)	52	121.34	2.33
**Total**	956	3836.76	4.01

[a]The average distance between the markes (cM/marker)
