# Supplementary material for: Uncovering the genetic basis of crown rust resistance in a northern-by-southern oat biparental population
Source: PLoS One. 2026 Jun 24;21(6):e0351420. doi: 10.1371/journal.pone.0351420 (PMC13293447; doi:10.1371/journal.pone.0351420)
Supplement: S4 Table — (PDF) [file pone.0351420.s004.pdf]

95% confidence interval of QTL identified in AIA1405 RIL population and their corresponding region in oat consensus map

		AIA1405 Linkage map	Oat consensus map[a]	
QTL	Chr	QTL confidence interval (cM)	QTL region (cM)	No. of markers
QPca-ars-2D	2D	132-149	89.9-99.8	161
QPca-ars-4A	4A	120-138	101.54-151.95	985
QPca-ars-7A[b]	7A	1-3	NA	NA
QPca-ars-7C	7C	89-93	80.86-89.66	533
QPca-ars-7D1	7D	113-117	29.14-29.80	75
QPca-ars-7D2	7D	114-119	29.40-30.31	

[a]The linkage group and position information was obtained from consensus map of Bekele et al. (2018)

[b]The markers within the Qpca-ars-7A region were not used in this analysis because of contrasting chromosomal location of markers between AIA1405 and consensus maps.
