# Supplementary material for: Uncovering the genetic basis of crown rust resistance in a northern-by-southern oat biparental population
Source: PLoS One. 2026 Jun 24;21(6):e0351420. doi: 10.1371/journal.pone.0351420 (PMC13293447; doi:10.1371/journal.pone.0351420)
Supplement: S5 Table — (PDF) [file pone.0351420.s005.pdf]

Marker-trait association results from analyses within fall-planted (primarily southern US) and spring-planted (northern) subsets of the CORE association mapping panel.

| Panels | Environment_Traits | Marker[a] | Chr | Pos | p-value | MarkerR2 |
|---|---|---|---|---|---|---|
| Northern | Winnipeg 2011_severity | ***avgbs_cluster_13926.1.38*** | 4A | 277.50 | 7.10E-07 | 0.07576 |
| | Winnipeg 2011_severity | ***avgbs_10324.1.31*** | 7C | 56.24 | 5.31E-06 | 0.07757 |
| | St Paul 2011_infection response | avgbs_63110.1.59 | 7D | 511.36 | 1.36E-04 | 0.04876 |
| | Fargo 2010_infection response | avgbs_63110.1.59 | 7D | 511.36 | 1.42E-04 | 0.07181 |
| Southern | Castroville 2010_severity | avgbs_6K_80511.1.64 | 4A | 275.84 | 7.24E-04 | 0.14002 |
| | Baton Rouge 2011_severity | avgbs_cluster_18331.1.38 | 4A | 298.63 | 7.55E-04 | 0.17322 |

[a]The markers in bold and italic are significant at bonferroni correction threshold while other markers are significant at $p<0.001$.
