# Supplementary material for: Uncovering the genetic basis of crown rust resistance in a northern-by-southern oat biparental population
Source: PLoS One. 2026 Jun 24;21(6):e0351420. doi: 10.1371/journal.pone.0351420 (PMC13293447; doi:10.1371/journal.pone.0351420)
Supplement: S6 Table — (PDF) [file pone.0351420.s006.pdf]

Effect of pyramiding resistance alleles on crown rust severity and infection response

Traits	Number of QTLs	RILs frequency	Mean	Relative Difference to null group (%)	Tukey's HSD ($p<0.05$)
Combined-SEV	0	15	54.72	X	A
	1	52	50.87	7.03	A
	2	51	46.84	14.40	B
	3	6	40.02	26.86	C
Combined-IR	0	24	0.84	X	A
	1	58	0.79	5.95	A
	2	38	0.71	15.48	B
