# Supplementary material for: Uncovering the genetic basis of crown rust resistance in a northern-by-southern oat biparental population
Source: PLoS One. 2026 Jun 24;21(6):e0351420. doi: 10.1371/journal.pone.0351420 (PMC13293447; doi:10.1371/journal.pone.0351420)
Supplement: S1 Fig — (PDF) [file pone.0351420.s007.pdf]

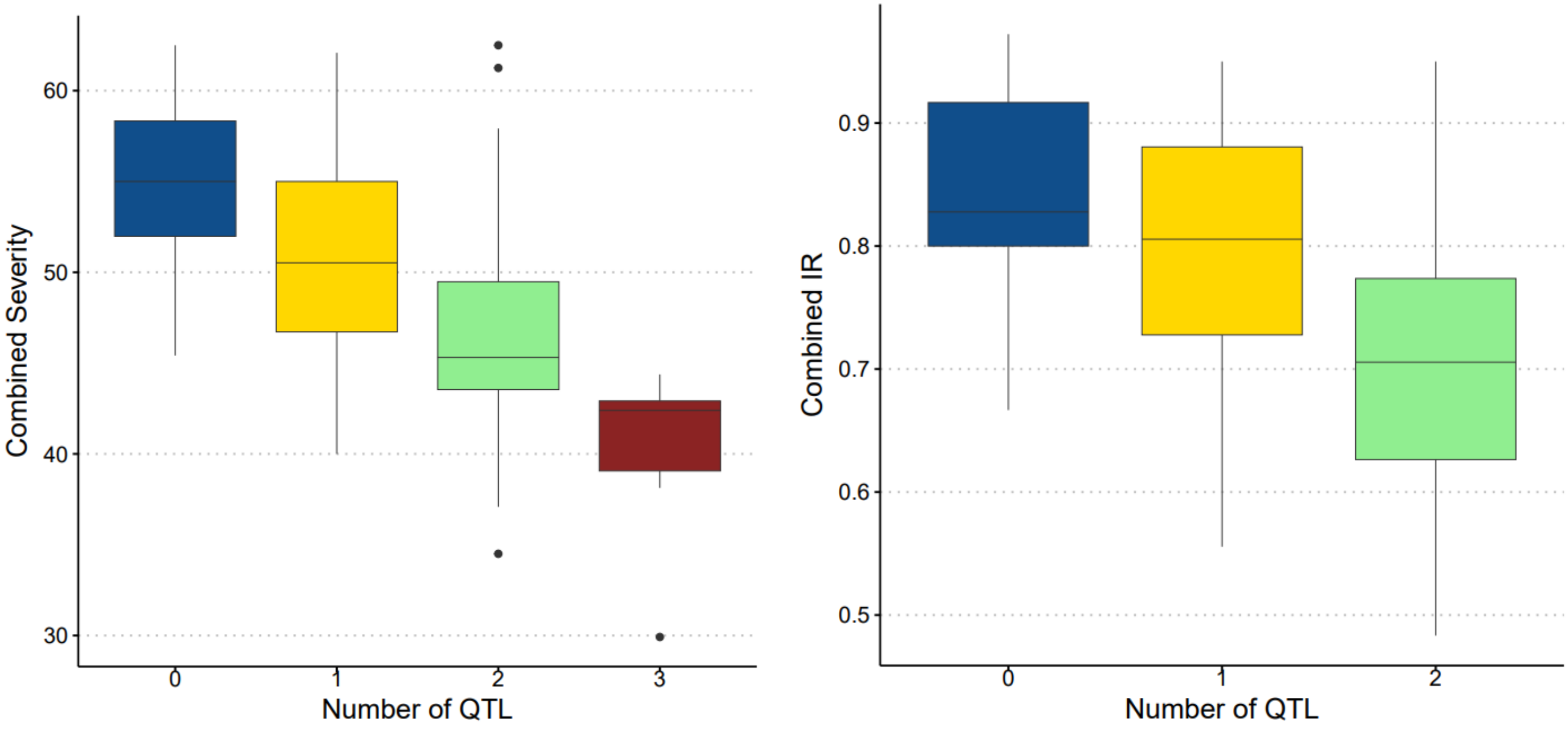

Figure S1: Boxplots demonstrating the effect of QTLs on crown rust severity (left) and IR (right) in the AIA1405 RIL population using the phenotypic data combined across all environments.
