# Supplementary material for: Uncovering the genetic basis of crown rust resistance in a northern-by-southern oat biparental population
Source: PLoS One. 2026 Jun 24;21(6):e0351420. doi: 10.1371/journal.pone.0351420 (PMC13293447; doi:10.1371/journal.pone.0351420)
Supplement: S2 Fig — (PDF) [file pone.0351420.s008.pdf]

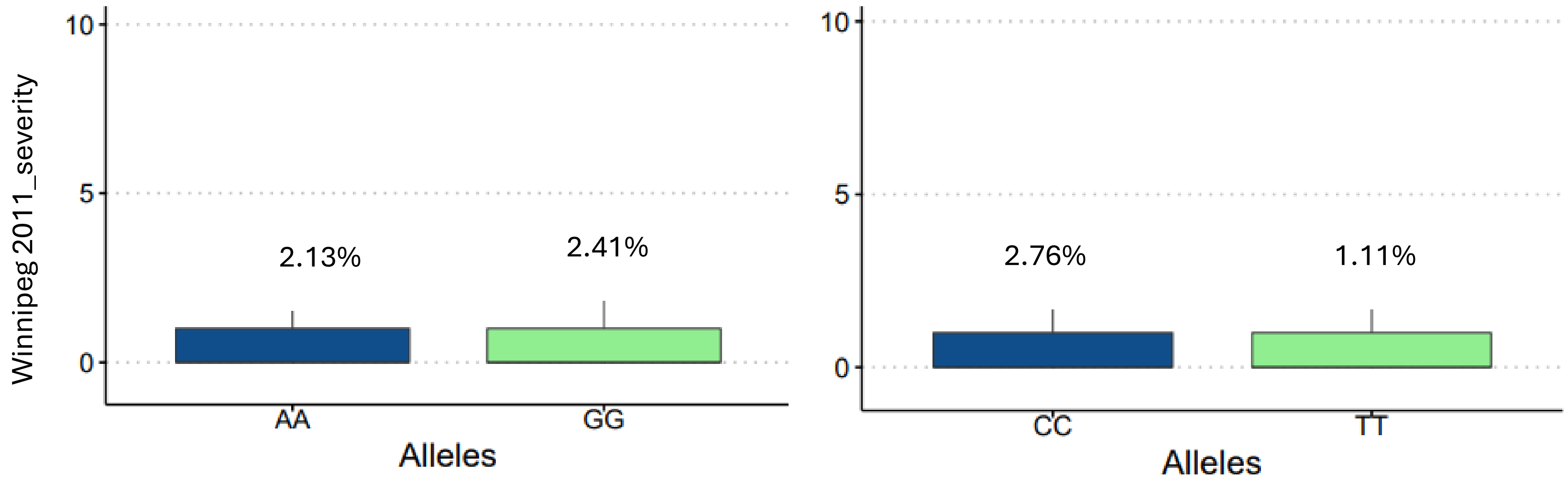

Figure S2: Distribution of crown rust severity data (Winnipeg 2011) of oat lines from Collaborative Oat Research Enterprise (CORE) panel carrying AA/GG and CC/TT genotypes at avgbs_10324.1.31(left) and avgbs_cluster_13926.1.38 (right) markers. The number above the boxplot represents the average data of lines carrying representative allele.
