# Supplementary material for: Uncovering the genetic basis of crown rust resistance in a northern-by-southern oat biparental population
Source: PLoS One. 2026 Jun 24;21(6):e0351420. doi: 10.1371/journal.pone.0351420 (PMC13293447; doi:10.1371/journal.pone.0351420)
Supplement: S3 Fig — (PDF) [file pone.0351420.s009.pdf]

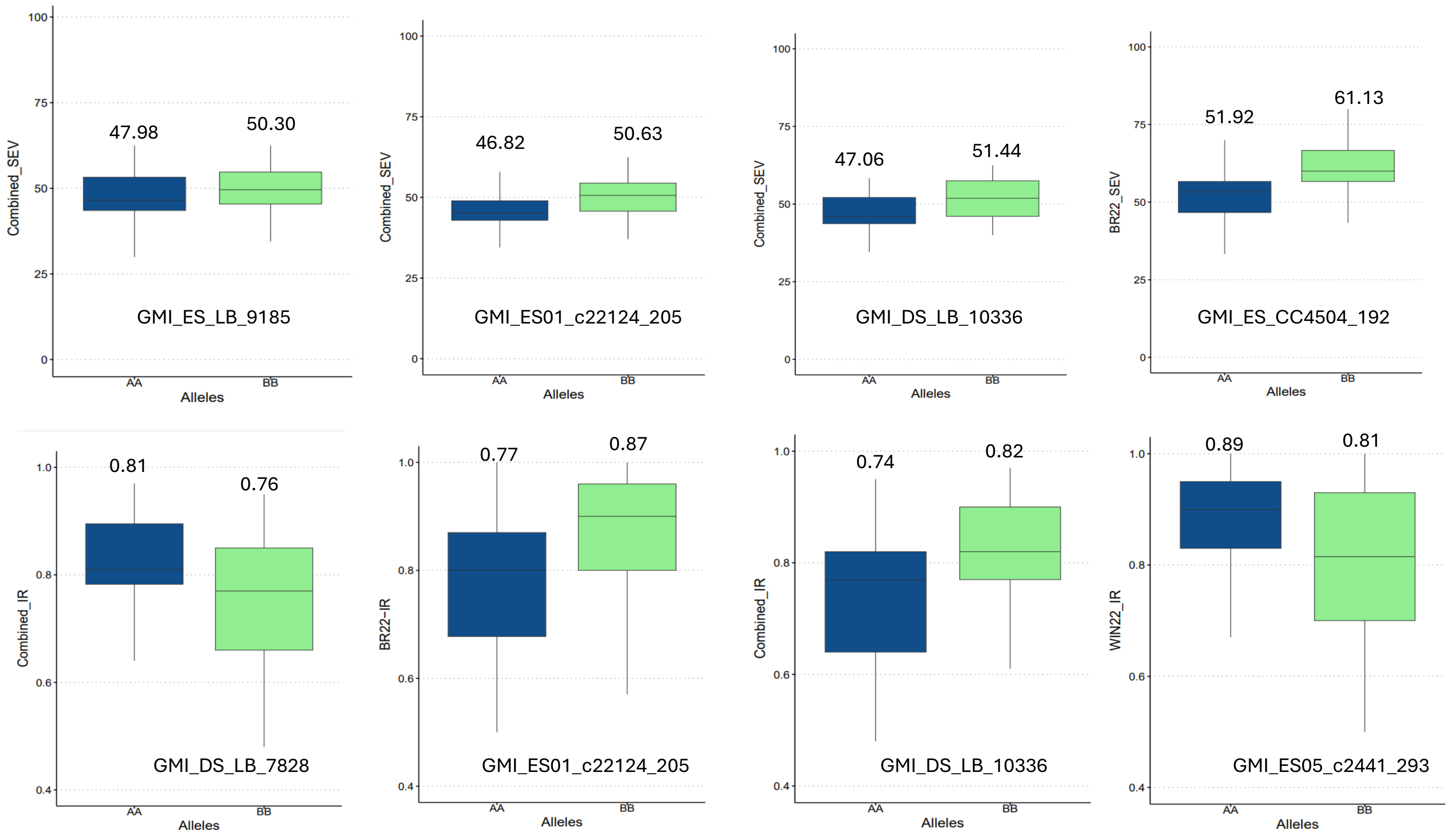

Figure S3: Phenotypic differences between homozygous genotype classes associated with six QTL in the AIA1405 recombinant inbred line population. The number above the boxplot represents the average data of lines carrying representative allele.
