# Supplementary material for: Uncovering the genetic basis of crown rust resistance in a northern-by-southern oat biparental population
Source: PLoS One. 2026 Jun 24;21(6):e0351420. doi: 10.1371/journal.pone.0351420 (PMC13293447; doi:10.1371/journal.pone.0351420)
Supplement: Attachment — Submitted filename: Response to reviewers.docx [file pone.0351420.s010.docx]

April 29, 2026

Re: PLOS ONE Decision: Revision required [PONE-D-26-08710]

Dear editor and reviewers,

Thank you very much for the review and valuable suggestions for further improvement of our manuscript “Uncovering the genetic basis of crown rust resistance in a northern-by-southern oat biparental population”. Please find attached our revised manuscript. As you can see, we have addressed all the points reviewers raised and hope we meet them to your satisfaction. The details of specific changes/edits are in the track-change format. If you require any additional information, please do not hesitate to contact me ([Kathy.klos@usda.gov](mailto:Kathy.klos@usda.gov)).

**Reviewer #1:**

This manuscript provides an analysis on the genetic basis of crown rust resistance in a bi-parental oat population, a useful endeavor to help breeders with the on-going battle against this disease. The manuscript is generally well-written and clear, with the data collection and analysis related to the bi-parental population done well. However, I don’t feel that the “Genotype-phenotype association analysis” part of the manuscript (using the southern and northern panels) makes a compelling case for the markers you identified or is supported by good data. I appreciate the attempt to validate the usefulness of the QTL identified, but I would not put too much trust in marker-phenotype association when disease severity observed in the Winnipeg 2011 nursery was <3%. This is a meaningless level of infection that would be highly subject to human error during rating and chance (in terms of infection establishment across the nursery). Aspects of the manuscript related to this work should be removed.

Response: We appreciate your suggestion to remove the genotype-phenotype association analysis part from the manuscript. The major goal of adding this part is to see if the QTL detected in this study using bi-parental population is also present in other diverse set of germplasm or not. We believe that although the result obtained from this analysis was not sufficient to validate all the QTL detected in this study, they still support, in part, our result and are able to validate two of six QTL detected. Also, another reviewer who reviewed this manuscript strongly supported this analysis. See comment from second reviewer below-

*“A significant strength of this work is the validation of detected QTL using independent oat sets (southern and northern CORE panels). This increases the likelihood that the identified markers will be effective across different germplasm”.*

Other edits and comments are provided below.

Ln 50: suggest “Increased awareness among consumers…”
(I think the awareness is more broadly among consumers, not oat consumers)

Response: Corrected.

Ln 60: if you are going to say that fungicide resistance is likely to develop, I think you need a reference showing this has occurred either in Puccinia coronata, or in other rusts of oat, barley or wheat.

Response: updated in the revised manuscript.

Ln 75: suggest “Under field evaluations at the adult plant stage, …”

Response: Corrected.

Ln 82-83: given that your manuscript relates to APR, I think it is necessary to indicate the location of those APR QTL that have been mapped, such as the APR identified in MN841801.

Response: The related information regarding APR QTL has been updated in the revised manuscript and three references are added.

Ln 85: suggest “… , especially to crown rust.” Also, I don’t think you can start a sentence with an acronym, need to spell out.

Response: Corrected.

Ln 103: I would disagree with the statement that CDC Dancer is susceptible, it is commonly rated Intermediate reaction to crown rust in Canada. Your QTL mapping would also indicate it carries some resistance.

Response: Thank you for your suggestion. We also agree that CDC Dancer showed some level of CR reaction which was evident from field scoring as well as QTL mapping in this study. We revised the manuscript as suggested.

Ln 113: please fix grammar in this sentence, specifically the “as head rows”

Response: corrected, thank you so much.

Ln 150: was there no criteria related to allele frequency? Would be good to note.

Response: The minor allele frequency information has been added in the revised manuscript.

Ln 162-164: Did you attempt to use a premutation test to determine significant LOD score?

Response: Yes, we performed permutation test to determine significant QTL in the QTL analysis. This info has been added in the revised manuscript.

Ln 180-182: Were these nurseries naturally infected and were any spreader rows included?

Response: The phenotypic data for the northern and southern panel were obtained from Klos et al. 2017 paper. This manuscript provides details about the location and how the crown rust phenotypic data was collected. We updated this reference in the paper.

*“Klos KE, Yimer BA, Babiker EM, Beattie AD, Bonman JM, Carson ML, et al. Genome‐wide association mapping of crown rust resistance in oat elite germplasm. The plant genome. 2017;10(2):plantgenome2016.10.0107*

Ln 198: “ There was not much…”

Response: Updated. Thank you!

Ln 198-202: you indicate that the population did not show much variation in disease response in BR21 but did in all the 2022 environments. In fact, there appears to be more variation in the BR21 environment than in the other three 2022 environments. Please alter this statement.

Response: Thank you for pointing out this error. We intended to talk about the variation in disease pressure. The initial year (BR21) had lower disease pressure, and therefore, most RILs showed LS severity <30%, however, the following year the disease pressure was higher. This information has been updated.

Ln 205: appears to be a typo at the end of this line

Response: Updated. Thank you!

Ln 207: suggest “…in Figure 1.”

Response: Updated. Thank you!

Ln 209: Table S1 is labelled S2 in the supplementary file.

Response: Corrected, thank you!

Ln 224: Table S2 is labelled S3 in the supplementary file.

Response: Corrected, thank you!

Ln 232: chromosome 1D also had 83 markers, you might want to mention. Also, Table S3 is labelled S4 in the supplementary file.

Response: Corrected, thank you!

Ln 229 (Genotyping data and linkage maps Section): I don’t think you need decimal places associated with the cM distances. You don’t have the marker density nor are you narrowing down a region (as per fine mapping) to justify that level of detail.

Response: Thank you so much for your suggestion. Yes, we agree that since this work is not focused on fine mapping, detail info about linkage maps is not required for general QTL mapping. However, to be consistent with our previous work, and to use these maps in the future (in case we decided to work on fine mapping of some QTL identified in this study), we decided to keep decimal point as it is.

Ln 252: this sentence should be more specific with respect to which LOD was associated with which trait. You should also mention the PVE for Combined-SEV.

Response: This suggestion has been incorporated in the revised manuscript.

Ln 258: I’m not sure why you consider the 2 QTL on 7D to be different QTL? Their 95% CIs are not mutually exclusive from one another so by your definition should be considered the same QTL. Please correct if you agree or explain why this would not be the case.

Response: Thank you for pointing out this. Yes, the substantial overlap in 1-LOD confidence intervals would be consistent with these peaks representing a single QTL. We have chosen to label them differently because there is evidence of linked or allelic effects contributed from both parents. We expand on this in the results and discussion sections.

Ln 262 (Validation of QTL using genotype-phenotype association Section): some explanation is required regarding why the 4A and 7C QTL were relevant in the Northern Panel when both alleles are derived from the southern parent (LA07065) which would not have been tested in Winnipeg.

Response: Thank you for this valuable suggestion. We have expanded on these analyses in the results and added some explanation on why QTL, mapped here from a southern line, were associated in the northern CORE panel. We added this information in the discussion section (please check 7^th^ paragraph of the discussion).

Ln 273 (QTL pyramiding effect): for the pyramiding of QTL related to combined severity, when you present the range in combined severity data for the 1 and 2 QTL situations, does this represent each of the 3 single QTL and all combinations of 2 QTL, respectively? Same question for the pyramiding of the IR QTL.

Response: For this analysis, we only selected the QTL that were associated with crown rust severity and IR data when the data from all environments was combined. For example, when we did the analysis for the combined severity data, there are three QTL (*QPca-ars-74A, QPca-ars-7A, and QPca-ars-7C)* that influenced combined crown rust severity data (see table 1). We used these three QTL and combined severity phenotypic data for this analysis. We classify the RIL based on number of QTL they carried (no QTL, 1 QTL, 2QTL, and 3QTL) and plotted their pheno data in the graph (supplementary figure 1). We also did similar analysis for IR QTL.

Ln 280: please add units to y-axis.

Response: We did not see a place to add unit in the y-axis at line 280, but if you are talking about supplementary figure, crown rust severity has been stated in the figure description (see supplementary figure 1 description).

Ln 302: I disagree with this statement, the 7C QTL was not detected consistently across environments, it was detected in one single environment and the combined environment. The combined environment is not equivalent to every single environment, and detection of the QTL in the combined environment may be largely driven by the BR-22 environment. Please change the wording of this sentence.

Response: Thank you for your suggestion. We deleted this statement in the revised manuscript.

Ln 310-111: “Ociepa and Okon [42] reported linkage maps in oat that covered a total genetic distance of
24,209.96 cM…” What does this mean? This number does not make sense with respect to the shortest and longest maps that you then report. If you are reporting the total length of all the maps in the reference you cite, then I don’t see how this is a relevant piece of information. Please remove.

Response: Thank you for your suggestion. We removed some part of this information in the revised manuscript. In our discussion we mentioned that the linkage map of oat developed in this study are comparable, and in some cases, represented an improvement to previously reported linkage maps in oat (see 2^nd^ paragraph of discussion). Therefore, we included this reference to show that previously reported map is larger than our map.

Ln 345-347: Although such genes are Pc48 are technically defeated, it is not possible that they are still influential in providing a small amount of resistance. Given that your QTL explain a limited amount of variation this would be in-line with such small influence of defeated genes. If I compare Canadian varieties (which undoubtedly contain defeated genes like Pc38, Pc68, Pc91 etc) rated as MS, they have a notably better level of resistance compared to many European varieties (a region where crown rust is not activity bred for) which carry no defeated Pc genes and would be rated VS (very susceptible).

Response: We also believe that the QTL detected on chromosome 4A represent *Pc48* based on evidence that they are close to each other (4-12 cM) and their overlapping position on GrainGenes OT3098 v2 QTL/gene inventory data (please look discussion 4^th^ paragraph). We have updated some information in our discussion section as –

“Although *Pc48* is defeated in many oat producing regions and fails to provide crown rust resistance [46], it is possible that *Pc48* was effective against some proportion of the naturally occurring crown rust in the environments used in this study, and contributed a small amount of resistance. ”

Ln 387-392: couple comments here, 1) this is the first time you are mentioning this data so it should be in the results section, 2) you mention rare alleles for both markers but do not indicate if these are the same alleles associated with resistance in the bi-parental mapping population, 3) how rare are they? If below 5% would that not make the results suspect?, 3) I don’t think I’d put too much trust in marker-phenotype association when disease severity was <3%. This is a meaningless level of infection, essentially none, that would be highly subject to human error during rating and chance (in terms of infection establishment across the nursery). No data was presented for the other markers in Table S5, so hard to make a judgement on the reliability of the marker-phenotype association in those cases.

I generally don’t feel that this part of the manuscript (i.e. the “Genotype-phenotype association analysis” using the southern and northern panels) makes a compelling case for the markers you identified or is supported by good data. I suggest removing this portion of the manuscript entirely. However, the information presented between lns 413-422 is useful and should be kept.

Response: Thank you so much for your valuable comments regarding genotype-phenotype association analysis part of the manuscript. An addition to the materials and methods section should clarify that only markers with at least 10 rare allele homozygotes were analyzed within a subpopulation (comment 3). Additional detail has been added to the result section relevant to the discussion (comment 1). This should clarify that markers most closely linked to the AIA1405 QTL were not available in the CORE (comment 2). We agree that mean field severity of 3% is very low and have added text to that effect in the discussion (comment 3).

We appreciate your suggestion to remove the genotype-phenotype association analysis part from the manuscript. However, we feel that this information provides context for the potential utility of the LA07065_BSBSBS_32.2-derived QTL in the wider North American breeding germplasm. Given that the CORE data, both genotype and phenotype, is publicly available, this discussion may also suggest a source of additional markers for use within specific breeding programs.

Ln 422-426: Again, this is the first time you have mentioned this data so it should be in the results section. Also, is there a statistical analysis to back up your statement regarding the 7D2 QTL marker? If not, please provide one or alter the statement.

Response: This section has been moved to result from the discussion. Please check “Detection of crown rust resistance QTL” section of the result.

Ln 428: this sentence is misleading as you are changing the frame of reference. The preceding studies you quote with respect to the economic impact of crown rust are using disease severity values as the unit of measure, but you report a 26% increase in resistance. Your 26% is based on a relative change in disease severity from 55% to 40%, the actual unit that matters is the 15% change in disease severity. Please alter this sentence to reflect this.

Response: Thank you for your suggestion. The changes have been made in the revised manuscript.

Ln 625 (Fig. 1): suggest that you include the units (i.e. %) on the y-axis label for Severity. I don’t think you need IR after Infection Response on the y-axis of the other boxplot figure. It would be helpful to show where the parental values fall out on each of the boxplots, perhaps with a single letter representing each parent placed on the boxplots?

Response: Thank you. The suggested information has been added in Figure 1 of the revised manuscript. Figure 1 was generated using the phenotypic data of the RIL only and parental lines were not added. For the phenotypic data of the parent, it has been mentioned in the result section (please check Phenotypic analysis of RIL population section of the result).

Ln 649 (Fig. 3): if the length of the bars next to the QTL mean anything then you should mention this in the caption. Also need to insert a space after (RIL) on the second line.

Response: Corrected. The length of the black bars next to the QTL does not mean anything except it is showing the location of QTL on the genetic map.

**Reviewer #2:**

It identifies novel QTL and validates them in diverse oat populations.
The statistical methods (ANOVA, heritability, ICIM-ADD mapping) and genotyping approaches are appropriate and well-documented. It provides actionable information (SNP markers) for oat breeders to improve disease resistance. The authors confirm that all underlying data are available within the manuscript and supporting files. The study employs a solid experimental design, including evaluations across four distinct field environments over two years. This multi-environment approach strengthens the reliability of the identified QTL. A significant strength of this work is the validation of detected QTL using independent oat sets (southern and northern CORE panels). This increases the likelihood that the identified markers will be effective across different germplasm.
The detection of a major, consistent QTL on chromosome 7C (QPca-ars-7C) that explains up to 16.54% of phenotypic variation provides a clear target for marker-assisted selection (MAS) in oat breeding programs. The research addresses a high-priority issue in oat production—crown rust—and identifies specific markers that can be used to develop cultivars with more durable, non-race-specific resistance. The manuscript follows standard genotyping and mapping protocols, utilizing the 6K Infinium iSelect SNP array and recognized software like QTL IciMapping and TASSEL.

Response: We appreciate your valuable time in reviewing this manuscript. Thank you for your suggestions/edits which further improve the quality of our work/manuscript.

The RIL population consists of 124 lines. While functional for biparental mapping, this is a relatively small population size by modern standards, which may limit the resolution of the genetic map and the ability to detect minor-effect QTL.

Response: We totally agree that number of RILs matters, especially when mapping genomic regions using linkage analysis. In this work, we started developing this population using >200 F2 progenies; however, as we continue advancing generations for homogeneity, we could not retain all lines that we started, and we ended up only with124 RILs for linkage analysis. Generally, when the size of mapping population is large, usually >150 lines, it helps to achieve robust QTL detection and decent resolution. However, we believe that in oat, 100-150 lines are acceptable for conducing linkage analysis. Please check references such as <https://doi.org/10.1094/PHYTO-97-9-1063> which used even less than 100 lines for conducting genetic analysis.

After quality filtering, only 956 polymorphic SNP markers were used for the linkage map. In the current era of high-density genotyping, this density is moderate and may leave some genomic regions underrepresented.
Response: We agree, but as you may know, unlike other cereals like wheat and barley, genotyping platform in oat is still developing. We believe that 956 high quality SNPs used in this study for linkage map construction and QTL mapping are sufficient for identifying genomic regions associated with crown rust. We have discussed this fact in the discussion section of the manuscript (please check 2^nd^ paragraph of the discussion).

The manuscript notes that disease pressure was relatively low in one environment (BR21). While multi-year data helps mitigate this, low-pressure environments can sometimes skew phenotypic averages.

Response: We agree. BR21 was not the best year for conducting evaluation nurseries for crown rust resistance. However, although the disease pressure was low, the population segregated well so that we used this environment in our analysis.

Although QPca-ars-7C is described as a "major" QTL, it explains 16.54% of the variation. While significant, this suggests that a large portion of the resistance is still controlled by many other unidentified minor loci or environmental factors.

Response: We agree on your point. We categories this QTL as “major” mainly based on two facts. Firstly, it explained 16.54% of phenotypic variation and secondly, it was consistently detected across multiple environments including the combined data set.
